# IARC Monographs: 40 Years of Evaluating Carcinogenic Hazards to Humans

**DOI:** 10.1289/ehp.1409149

**Published:** 2015-02-24

**Authors:** Neil Pearce, Aaron Blair, Paolo Vineis, Wolfgang Ahrens, Aage Andersen, Josep M. Anto, Bruce K. Armstrong, Andrea A. Baccarelli, Frederick A. Beland, Amy Berrington, Pier Alberto Bertazzi, Linda S. Birnbaum, Ross C. Brownson, John R. Bucher, Kenneth P. Cantor, Elisabeth Cardis, John W. Cherrie, David C. Christiani, Pierluigi Cocco, David Coggon, Pietro Comba, Paul A. Demers, John M. Dement, Jeroen Douwes, Ellen A. Eisen, Lawrence S. Engel, Richard A. Fenske, Lora E. Fleming, Tony Fletcher, Elizabeth Fontham, Francesco Forastiere, Rainer Frentzel-Beyme, Lin Fritschi, Michel Gerin, Marcel Goldberg, Philippe Grandjean, Tom K. Grimsrud, Per Gustavsson, Andy Haines, Patricia Hartge, Johnni Hansen, Michael Hauptmann, Dick Heederik, Kari Hemminki, Denis Hemon, Irva Hertz-Picciotto, Jane A. Hoppin, James Huff, Bengt Jarvholm, Daehee Kang, Margaret R. Karagas, Kristina Kjaerheim, Helge Kjuus, Manolis Kogevinas, David Kriebel, Petter Kristensen, Hans Kromhout, Francine Laden, Pierre Lebailly, Grace LeMasters, Jay H. Lubin, Charles F. Lynch, Elsebeth Lynge, Andrea ‘t Mannetje, Anthony J. McMichael, John R. McLaughlin, Loraine Marrett, Marco Martuzzi, James A. Merchant, Enzo Merler, Franco Merletti, Anthony Miller, Franklin E. Mirer, Richard Monson, Karl-Cristian Nordby, Andrew F. Olshan, Marie-Elise Parent, Frederica P. Perera, Melissa J. Perry, Angela Cecilia Pesatori, Roberta Pirastu, Miquel Porta, Eero Pukkala, Carol Rice, David B. Richardson, Leonard Ritter, Beate Ritz, Cecile M. Ronckers, Lesley Rushton, Jennifer A. Rusiecki, Ivan Rusyn, Jonathan M. Samet, Dale P. Sandler, Silvia de Sanjose, Eva Schernhammer, Adele Seniori Costantini, Noah Seixas, Carl Shy, Jack Siemiatycki, Debra T. Silverman, Lorenzo Simonato, Allan H. Smith, Martyn T. Smith, John J. Spinelli, Margaret R. Spitz, Lorann Stallones, Leslie T. Stayner, Kyle Steenland, Mark Stenzel, Bernard W. Stewart, Patricia A. Stewart, Elaine Symanski, Benedetto Terracini, Paige E. Tolbert, Harri Vainio, John Vena, Roel Vermeulen, Cesar G. Victora, Elizabeth M. Ward, Clarice R. Weinberg, Dennis Weisenburger, Catharina Wesseling, Elisabete Weiderpass, Shelia Hoar Zahm

**Affiliations:** 1 Department of Medical Statistics, London School of Hygiene and Tropical Medicine, London, United Kingdom; 2 Division of Cancer Epidemiology and Genetics, National Cancer Institute (NCI), National Institutes of Health (NIH), Department of Health and Human Services (DHHS), Bethesda, Maryland, USA; 3 Imperial College, London, United Kingdom; 4 Leibniz Institute for Prevention Research and Epidemiology – BIPS, Bremen, Germany; 5 Department of Research, Cancer Registry of Norway, Oslo, Norway; 6 Centre for Research in Environmental Epidemiology (CREAL), IMIM (Hospital del Mar Medical Research Institute), Universitat Pompeu Fabra (UPF), and CIBER Epidemiologia y Salud Publica (CIBERESP), Barcelona, Spain; 7 School of Public Health, The University of Sydney and Sax Institute, Sydney, New South Wales, Australia; 8 Department of Environmental Health, and Department of Epidemiology, Harvard T.H. Chan School of Public Health, Boston, Massachusetts, USA; 9 Division of Biochemical Toxicology, National Center for Toxicological Research, Jefferson, Arkansas, USA; 10 Department of Clinical Sciences and Community Health, University of Milan and IRCCS Foundation Ca’Granda Ospedale Maggiore Policlinico, Milan, Italy; 11 NCI, NIH, DHHS, Research Triangle Park, North Carolina, USA; 12 Division of Public Health Sciences, and Alvin J. Siteman Cancer Center, Washington University, School of Medicine, St. Louis, Missouri, USA; 13 Division of the National Toxicology Program, National Institute of Environmental Health Sciences (NIEHS), NIH, DHHS, Research Triangle Park, North Carolina, USA; 14 KP Cantor Environmental LLC, Silver Spring, Maryland, USA; 15 CREAL, IMIM, and CIBERESP, Barcelona, Spain; 16 Institute of Occupational Medicine, Edinburgh, United Kingdom; 17 Department of Public Health, Clinical and Molecular Medicine, University of Cagliari-Monserrato, Cagliari, Italy; 18 MRC Lifecourse Epidemiology Unit, University of Southampton, Southampton, United Kingdom; 19 Department of Biology and Biotechnology, “Charles Darwin” Sapienza Rome University, Rome, Italy; 20 Occupational Cancer Research Centre, Cancer Care Ontario, Toronto, Ontario, Canada; 21 Division of Occupational and Environmental Medicine, Duke University Medical Center, Durham, North Carolina, USA; 22 Centre for Public Health Research, Massey University, Wellington, New Zealand; 23 Department of Environmental Health Science, and Department of Epidemiology, School of Public Health, University of California, Berkeley, Berkeley, California, USA; 24 Department of Epidemiology, University of North Carolina at Chapel Hill, Chapel Hill, North Carolina, USA; 25 Department of Environmental and Occupational Health Sciences, University of Washington School of Public Health, Seattle, Washington, USA; 26 European Centre for Environment and Human Health, University of Exeter Medical School, Truro, Cornwall, United Kingdom; 27 Department of Social and Environmental Health Research, London School of Hygiene and Tropical Medicine, London, United Kingdom; 28 Louisiana State University School of Public Health, New Orleans, Louisiana, USA; 29 Department of Epidemiology, Lazio Regional Health Service, Rome, Italy; 30 Center for Environmental Research and Sustainable Technology (UFT), Universität Bremen, Bremen, Germany; 31 School of Public Health, Curtin University, Perth, Western Australia, Australia; 32 Department of Environmental and Occupational Health, Ecole de Santé Publique, Universite de Montreal, Montreal, Quebec, Canada; 33 Population-based Epidemiological Cohorts Unit, Inserm UMS 011, Villejuif, France; 34 Department of Environmental Medicine, University of Southern Denmark, Odense, Denmark; 35 Institute of Environmental Medicine (IMM), Karolinska Institutet, Stockholm, Sweden; 36 Danish Cancer Society Research Center, Copenhagen, Denmark; 37 Department of Epidemiology and Biostatistics, Netherlands Cancer Institute, Amsterdam, the Netherlands; 38 Division of Environmental Epidemiology, Institute for Risk Assessment Sciences, Utrecht University, Utrecht, the Netherlands; 39 German Cancer Research Center (DKFZ), Heidelberg, Germany; 40 Epidemiology and Biostatistics Sorbonne Paris Cite Center (CRESS), INSERM, UMR 1153, Epidemiology of Childhood and Adolescent Cancers Research Group (EPICEA), Paris Descartes University, F-75015, Paris, France; 41 Department of Public Health Sciences, University of California, Davis, Davis, California, USA; 42 Department of Biological Sciences, North Carolina State University, Raleigh, North Carolina, USA; 43 NIEHS, NIH, DHHS, Research Triangle Park, North Carolina, USA; 44 Department of Public Health and Clinical Medicine, Umea University, Umea, Sweden; 45 College of Medicine, Seoul National University, Seoul, Korea; 46 Geisel School of Medicine at Dartmouth, Hanover, New Hampshire, USA; 47 National Institute of Occupational Health, Oslo, Norway; 48 CREAL, IMIM, and CIBERESP, Barcelona, Spain; National School of Public Health, Athens Greece; 49 Department of Work Environment, University of Massachusetts Lowell, Lowell, Massachusetts, USA; 50 Centre Francois Baclesse, Universite de Caen, Caen, France; 51 Department of Environmental Health, University of Cincinnati College of Medicine, Cincinnati, Ohio, USA; 52 Department of Epidemiology, College of Public Health, University of Iowa, Iowa City, Iowa, USA; 53 Department of Public Health, University of Copenhagen, Copenhagen, Denmark; 54 National Centre for Epidemiology and Population Health, Australian National University, Canberra, Australian Capital Territory, Australia; 55 Public Health Ontario, Toronto, Ontario, Canada; 56 Prevention and Cancer Control, Cancer Care Ontario, and Dalla Lana School of Public Health, University of Toronto, Toronto, Ontario, Canada; 57 World Health Organization Regional Office for Europe, European Centre for Environment and Health, Bonn, Germany; 58 Regional Mesothelioma Register, National Health Service, Local Health Authority, Padova, Italy; 59 Unit of Cancer Epidemiology, Department of Medical Sciences, University of Turin, Turin, Italy; 60 Dalla Lana School of Public Health, University of Toronto, Toronto, Ontario, Canada; 61 City University of New York (CUNY) School of Public Health, New York, New York, USA; 62 INRS-Institut Armand-Frappier, Universite du Quebec, Laval, Quebec, Canada; 63 Department of Environmental Health Sciences, and Columbia Center for Children’s Environmental Health, Mailman School of Public Health, Columbia University, New York, New York, USA; 64 Department of Environmental and Occupational Health, George Washington University Milken Institute School of Public Health, Washington, DC, USA; 65 IMIM, CIBERESP, and School of Medicine, Universitat Autònoma de Barcelona, Barcelona, Spain; 66 Finnish Cancer Registry, Institute for Statistical and Epidemiological Cancer Research, Helsinki, Finland, and School of Health Sciences, University of Tampere, Tampere, Finland; 67 Department of Environmental Health, University of Cincinnati College of Medicine, Cincinnati, Ohio, USA; 68 School of Environmental Sciences, Ontario Agricultural College, University of Guelph, Guelph, Ontario, Canada; 69 Department of Epidemiology, Fielding School of Public Health, University of California at Los Angeles, Los Angeles, California, USA; 70 Department of Pediatric Oncology, Emma Children’s Hospital/Academic Medical Center, Amsterdam, the Netherlands; 71 MRC-PHE Centre for Environment and Health, Department of Epidemiology and Biostatistics, Imperial College London, London, United Kingdom; 72 Department of Preventive Medicine and Biometrics, Uniformed Services University of the Health Sciences, Bethesda, Maryland, USA; 73 Department of Veterinary Integrative Biosciences, Texas A&M University, College Station, Texas, USA; 74 Department of Preventive Medicine, Keck School of Medicine, University of Southern California, Los Angeles, California, USA; 75 Epidemiology Branch, NIEHS, NIH, DHHS, Research Triangle Park, North Carolina, USA; 76 Cancer Epidemiology Research Programme, Unit of Infections and Cancer, Bellvitge Biomedical Research Institute (IDIBELL), CIBERESP Catalan Institute of Oncology, Barcelona, Spain; 77 Cancer Prevention and Research Institute (ISPO), Florence, Italy; 78 Department of Social and Preventive Medicine, Ecole de Santé Publique, Universite de Montreal, Montreal, Quebec, Canada; 79 Laboratory of Public Health and Population Studies, Department of Molecular Medicine, University of Padova, Padova, Italy; 80 The School of Public Health, University of California, Berkeley, Berkeley, California, USA; 81 Environmental Health Sciences, School of Public Health, University of California, Berkeley, Berkeley, California, USA; 82 Cancer Control Research, BC Cancer Agency and School of Population and Public Health, University of British Columbia, Vancouver, British Columbia, Canada; 83 Department of Molecular and Cellular Biology, Baylor College of Medicine, Houston, Texas, USA; 84 Department of Psychology, Colorado State University, Fort Collins, Colorado, USA; 85 Division of Epidemiology and Biostatistics, University of Illinois at Chicago, School of Public Health, Chicago, Illinois, USA; 86 Department of Environmental Health, Rollins School of Public Health, Emory University, Atlanta, Georgia, USA; 87 Exposure Assessment Applications LLC, Arlington, Virginia, USA; 88 Cancer Control Program, South East Sydney Public Health Unit, Randwick, New South Wales, Australia; 89 Stewart Exposure Assessments LLC, Arlington, Virginia, USA; 90 Department of Epidemiology, Human Genetics and Environmental Sciences, University of Texas School of Public Health, Houston, Texas, USA; 91 University of Torino and Centro di Riferimento per l’Epidemiologia e la Prevenzione Oncologica, CPO Piemonte, Torino, Italy; 92 Finnish Institute of Occupational Health, Helsinki, Finland; 93 Department of Public Health Sciences, Medical University of South Carolina, Charleston, South Carolina, USA; 94 Universdade Federal de Pelotas, Rio Grande do Sul, Brazil; 95 American Cancer Society Inc., Atlanta, Georgia, USA; 96 Biostatistics and Computational Biology Branch, NIEHS, NIH, DHHS, Research Triangle Park, North Carolina, USA; 97 Department of Pathology, City of Hope National Medical Center, Duarte, California, USA; 98 Institute of Environmental Medicine, Karolinska Institutet, Stockholm, Sweden; 99 Department of Community Medicine, Faculty of Health Sciences, UiT the Arctic University of Norway, Tromsø, Norway; Cancer Registry of Norway, Oslo, Norway; Genetic Epidemiology Group, Folkhalsan Research Center, Helsinki, Finland; Department of Medical Epidemiology and Biostatistics, Karolinska Institutet, Stockholm, Sweden; 100 Shelia Zahm Consulting, Hermon, Maine, USA; * Deceased

## Abstract

Background: Recently, the International Agency for Research on Cancer (IARC) Programme for the Evaluation of Carcinogenic Risks to Humans has been criticized for several of its evaluations, and also for the approach used to perform these evaluations. Some critics have claimed that failures of IARC Working Groups to recognize study weaknesses and biases of Working Group members have led to inappropriate classification of a number of agents as carcinogenic to humans.

Objectives: The authors of this Commentary are scientists from various disciplines relevant to the identification and hazard evaluation of human carcinogens. We examined criticisms of the IARC classification process to determine the validity of these concerns. Here, we present the results of that examination, review the history of IARC evaluations, and describe how the IARC evaluations are performed.

Discussion: We concluded that these recent criticisms are unconvincing. The procedures employed by IARC to assemble Working Groups of scientists from the various disciplines and the techniques followed to review the literature and perform hazard assessment of various agents provide a balanced evaluation and an appropriate indication of the weight of the evidence. Some disagreement by individual scientists to some evaluations is not evidence of process failure. The review process has been modified over time and will undoubtedly be altered in the future to improve the process. Any process can in theory be improved, and we would support continued review and improvement of the IARC processes. This does not mean, however, that the current procedures are flawed.

Conclusions: The IARC Monographs have made, and continue to make, major contributions to the scientific underpinning for societal actions to improve the public’s health.

Citation: Pearce N, Blair A, Vineis P, Ahrens W, Andersen A, Anto JM, Armstrong BK, Baccarelli AA, Beland FA, Berrington A, Bertazzi PA, Birnbaum LS, Brownson RC, Bucher JR, Cantor KP, Cardis E, Cherrie JW, Christiani DC, Cocco P, Coggon D, Comba P, Demers PA, Dement JM, Douwes J, Eisen EA, Engel LS, Fenske RA, Fleming LE, Fletcher T, Fontham E, Forastiere F, Frentzel-Beyme R, Fritschi L, Gerin M, Goldberg M, Grandjean P, Grimsrud TK, Gustavsson P, Haines A, Hartge P, Hansen J, Hauptmann M, Heederik D, Hemminki K, Hemon D, Hertz-Picciotto I, Hoppin JA, Huff J, Jarvholm B, Kang D, Karagas MR, Kjaerheim K, Kjuus H, Kogevinas M, Kriebel D, Kristensen P, Kromhout H, Laden F, Lebailly P, LeMasters G, Lubin JH, Lynch CF, Lynge E, ‘t Mannetje A, McMichael AJ, McLaughlin JR, Marrett L, Martuzzi M, Merchant JA, Merler E, Merletti F, Miller A, Mirer FE, Monson R, Nordby KC, Olshan AF, Parent ME, Perera FP, Perry MJ, Pesatori AC, Pirastu R, Porta M, Pukkala E, Rice C, Richardson DB, Ritter L, Ritz B, Ronckers CM, Rushton L, Rusiecki JA, Rusyn I, Samet JM, Sandler DP, de Sanjose S, Schernhammer E, Seniori Costantini A, Seixas N, Shy C, Siemiatycki J, Silverman DT, Simonato L, Smith AH, Smith MT, Spinelli JJ, Spitz MR, Stallones L, Stayner LT, Steenland K, Stenzel M, Stewart BW, Stewart PA, Symanski E, Terracini B, Tolbert PE, Vainio H, Vena J, Vermeulen R, Victora CG, Ward EM, Weinberg CR, Weisenburger D, Wesseling C, Weiderpass E, Zahm SH. 2015. IARC Monographs: 40 years of evaluating carcinogenic hazards to humans. Environ Health Perspect 123:507–514; http://dx.doi.org/10.1289/ehp.1409149

## Introduction

Important advances in human health have come from the recognition of health hazards and the development of policy actions to address them (Brownson et al. 2009; Espina et al. 2013; Samet 2000). Government and nongovernmental organizations use expert panels to review the scientific literature and to assess its relevance to public health policies. Scientific experts are charged with reviewing the quality and quantity of the scientific evidence and providing scientific interpretations of the evidence that underpin a range of health policy decisions.

The *IARC Monographs on the Evaluation of Carcinogenic Risks to Humans* of the International Agency for Research on Cancer (IARC) are a prominent example of such an expert review process. The goal of the Monograph Programme is to assess carcinogenic hazards from occupational, environmental, and lifestyle exposures and agents, thus providing an essential step in the societal decision-making process to identify and then control carcinogenic hazards. For these evaluations, IARC assembles groups of scientists with a range of relevant scientific expertise (called “Working Groups”) to review and assess the quality and strength of evidence from informative publications and perform a hazard evaluation to assess the likelihood that the agents of concern pose a cancer hazard to humans (Tomatis 1976). IARC has used this approach for four decades, since the first Monograph in 1972 (IARC 1972). Although widely accepted internationally, there have been criticisms of the classification of particular agents in the past, and more recent criticisms have been directed at the general approach adopted by IARC for such evaluations (Boffetta et al. 2009; Epidemiology Monitor 2012; Ioannidis 2005; Kabat 2012; McLaughlin et al. 2010, 2011).

The Monographs are widely used and referenced by governments, organizations, and the public around the world; therefore, it is critical that Working Group conclusions be clear and transparent. In addition to the actual evaluation, a major contribution of the Monographs is the assembly of relevant literature and its dissemination to the public. We recognize that no system of evaluation is perfect. It is important to foster continuing improvement of the methods used by IARC and other bodies that review scientific evidence. The IARC process itself has been modified from time to time (e.g., addition of specific evaluation of mechanistic data and greater use of formal meta-analyses and data-pooling approaches). Indeed, as recently as April 2014, the IARC Monographs program has been a subject of a review by the Advisory Group to recommend priorities for IARC Monographs during 2015–2019 (Straif et al. 2014). The Advisory Group has made a number of recommendations on further improvements in the Monographs process specifically related to conflict of interest, transparency, and the use of the systematic review procedures in data gathering and evaluation. Thus, possible changes to the process are periodically considered by IARC governing groups (Scientific Council and Governing Council) and Advisory Groups.

Here, we focus on current IARC processes and practices because these have been the focus of recent criticisms. The authors of this Commentary are scientists from a wide range of disciplines who are involved in designing and conducting studies that provide data used in hazard evaluations, such as those performed by IARC. Many (but not all) of us have served on IARC Monograph Working Groups, but none are current IARC staff. We first discuss the history of IARC, and describe how the IARC evaluations are performed in order to foster evidence-based policy. We then describe why unbiased evaluations, based on the evidence and free of conflicts of interest, are necessary for public health decision making. Finally, we discuss the recent criticisms of the IARC approach.

## The IARC Monographs

*History of the IARC Monographs.* Shortly after IARC’s establishment, its parent entity, the World Health Organization (WHO), asked IARC to prepare a list of agents known to cause cancer in humans. IARC recognized the need for a systematic process to determine which agents should be listed. Such a process was launched in 1972 by Lorenzo Tomatis, then Chief of the Division of Carcinogenicity of IARC (Tomatis 1976). IARC is funded by the governments of 24 countries that have decided to become members, in addition to competitive grants from funding agencies. The IARC Monograph Programme is mainly funded by the U.S. National Cancer Institute through a renewable grant subject to peer review of the program. Other sources of external funding have included the European Commission Directorate-General of Employment, Social Affairs and Equal Opportunities; the U.S. National Institute of Environmental Health Sciences; and the U.S. Environmental Protection Agency.

The IARC process antedates current systematic review methods, but anticipated some of them, for example, with regard to transparent literature identification. In the IARC process, agents are assessed for carcinogenic hazard and assigned to one of five categories, ranging from carcinogenic to humans to probably not carcinogenic to humans (Appendix 1). The classification categories are described in the preamble to the Monographs (IARC 2006). Carcinogenic hazard identification refers to an assessment of whether an agent causes cancer. Hazard identification does not predict the magnitude of cancer risks under specific conditions; this can be determined only with appropriate exposure–response information (National Research Council 2009).

*The IARC Monograph process*. The process for the preparation of an IARC Monograph is clearly described in the Preamble, which is published as part of each Monograph (e.g., IARC 2014a). It starts with the nomination of candidate agents. Nominations come from national regulatory agencies, scientists, and stakeholders, including public health professionals, experts in environmental or occupational hygiene, industry representatives, and private citizens. It is important to note that anyone (including private citizens) can participate in the nomination process. The Monograph Programme convenes meetings of special Advisory Groups (composed of external scientists that possess a broad range of relevant professional skills) to review agents nominated for evaluation and to suggest IARC priorities for such reviews (Ward et al. 2010). Announcements of a review are made on the IARC website (http://monographs.iarc.fr/ENG/Meetings/). For example, in 2013 IARC sought nominations for agents to be evaluated in 2015–2019 (IARC 2014b). An Advisory Group reviewed the nominated agents and exposures, added several new ones, and discussed the priorities for each.

The IARC staff makes the final selection of agents for review by taking into account the prevalence and intensity of exposure (of both occupational groups and the general population) and availability of sufficient literature for an evaluation of carcinogenicity, as well as advice from the Advisory Groups. The large majority of evaluations concern specific compounds, but there are also monographs on various occupations or industries, for example, aluminum production, insecticide applicators, firefighters, manufacture of leather goods, leather tanning and processing, welding, painters, petroleum refining, and pulp and paper manufacturing. Some individual exposures that occur in these settings have also been evaluated.

The next step is the selection of members of the Working Group (WG). IARC staff review the literature to identify Working Group candidates and specialists in relevant areas of expertise; they also seek names of possible candidates from the scientific community and advisory groups. The list of potential members, including disclosure of relevant conflicts of interest, is posted on the IARC website (http://monographs.iarc.fr/ENG/Meetings/) before the WG is convened, and anyone can send comments. Members are typically scientists who have conducted research relevant to the agent under review, but not necessarily on the specific agent. Selection procedures are evaluated yearly by the Scientific and the Governing Councils. The IARC Section of Monographs also has an external Advisory Board, made up of independent scientists, that periodically peer reviews its activities. In addition to Working Group members, invited specialists, representatives of health agencies, stakeholder observers, and the IARC Secretariat also attend meetings.

The responsibility of the Working Group is to review the literature before the Monograph meeting, discuss the literature at the meeting, and then classify whether an agent is carcinogenic, probably carcinogenic, possibly carcinogenic, not classifiable, or probably not carcinogenic to humans (see Appendix 1). Working Group members are also responsible for writing the IARC Monograph, which must both review the literature and explain why the Working Group came to their specific conclusions.

The procedures used to evaluate the scientific evidence are described in the Preamble to the Monographs (IARC 2006). It is important to stress that only Working Group members conduct the actual evaluation (Wild and Cogliano 2011; Wild and Straif 2011). IARC staff facilitate the evaluation process and ensure that the procedures described in the Preamble are followed; however, they do not determine the outcomes.

IARC assessments of carcinogenicity are based on, and necessarily limited to, scientific evidence available at the time of the review. The evidence comes from epidemiologic studies, animal bioassays, pharmacokinetic/mechanistic experiments, and surveys of human exposure. The aim is to include all relevant papers on cancer in humans and experimental animals that have been published, or accepted for publication, in peer-reviewed scientific journals and also any publicly available government or agency documents that provide data on the circumstances and extent of human exposure. To that end, the search of the literature takes a comprehensive approach. Papers that are found not to provide useful evidence can be excluded later in the process. IARC staff first use previous IARC Monographs (if available), database searches using relevant text strings, and contact with investigators in the field to identify potentially relevant material. Thus, the initial assembly of the literature is performed by individuals who are not engaged in the actual evaluation. Working Group members are then assigned various writing tasks and are instructed to perform their own literature searches to identify any further papers that might have been missed. In addition, all of the papers assembled by IARC are made available to the full Working Group before they meet, and any member can recommend other papers not previously identified that they think should be considered. Finally, papers can be recommended by stakeholder representatives before or during the Working Group meeting.

At the meeting of the Working Group, the assembled documents are reviewed and summarized by discipline-related subgroups.However, any member of the Working Group has access to all of the assembled literature. The summaries are distributed to all subgroups, and information from all disciplines is discussed in plenary sessions prior to assigning the agents to a specific carcinogenicity category.

Because new findings continually emerge in the literature, agents are reconsidered when IARC and IARC Advisory Groups judge that there is sufficient additional information that might alter a previous evaluation. Thus, conclusions regarding human carcinogenicity of particular substances may change as new evidence becomes available. For some agents, this reevaluation has resulted in progression toward greater certainty regarding their human carcinogenicity, whereas for others the progress has been moved toward less certainty. Such movements are expected in an open, transparent, and evidence-based process. A comprehensive update of all Group 1 carcinogens was recently accomplished in Volume 100 A through F (http://monographs.iarc.fr/ENG/Monographs/PDFs/index.php).

Usually, several agents are evaluated in a single meeting lasting more than 1 week. After discussing the evidence fully, the Working Group members follow the published IARC procedures for combining information from epidemiologic studies and bioassays to arrive at a preliminary classification (IARC 2014a). Mechanistic data are then considered in order to determine whether they warrant a change from the preliminary classification. The Working Group then votes on the final determination. Many votes are unanimous, but on occasion some reviewers may favor a higher or lower ranking than the majority. When there is dissent, alternative interpretations and their underlying reasoning are sometimes reported in the rationale for the evaluation if the dissenters feel their point of view is not sufficiently addressed in the monograph.

*Consideration of the totality of the evidence*. IARC Working Groups make every effort to provide full and transparent documentation of what evidence was assembled, how it was evaluated, and which papers were most important for the hazard evaluation. Consequently, the monographs are often quite lengthy, containing many evidence tables [see, for example, the recent monograph on trichloroethylene (IARC 2014c)]. Evaluations involve consideration of all of the known relevant evidence from epidemiologic, animal, pharmacokinetic/mechanistic, and exposure studies to assess cancer hazard in humans. Information on human exposure is not formally graded as part of the overall assessment of carcinogenic hazard; however, these data make a critical contribution to the process by characterizing the timing, duration, and levels of exposure in the population, and in evaluating the quality of the exposure assessment in epidemiologic studies.

Doubts and criticisms have sometimes been expressed about the relative weights attributed to evidence from individual disciplines to the assessment of cancer hazards to humans; however, each discipline provides important evidence toward the overall evaluation of causality according to the Bradford Hill considerations (Hill 1965). Because the totality of the evidence is considered, deficiencies in one discipline are often offset by strengths in another. For example, epidemiologic studies may focus on population-relevant exposures, whereas findings from animal experiments usually involve higher exposures but are less susceptible to confounding.

Long-term animal bioassays and mechanistic studies provide critical information on the capacity of an agent to produce cancer in mammalian systems, including humans, and to contribute to decisions that would lead to better protection of human health. Bioassays are the backbone of regulatory science because they provide the opportunity to rigorously evaluate potential hazards before there is widespread human exposure. Bioassays and mechanistic studies are sometimes criticized for employing exposure routes and doses that in most instances humans would not experience, although experimental dose categories sometimes approach exposure levels found in occupational situations. There is evidence that carcinogenicity in human and animal studies is often concordant, although data may differ as to the affected cancer site (Haseman 2000; Maronpot et al. 2004; Tomatis 2002). A major effort to evaluate the concordance between animal and human results is currently under way; two Working Groups were convened at IARC in 2012, and a systematic evaluation of the correspondence between human and animal data was undertaken (a report is not yet publicly available).

## Criticisms of the IARC Process

IARC Monographs are widely used to identify potential carcinogenic hazards to humans and serve as reference documents summarizing the literature on many different agents. In recent years, however, individuals have criticized both the classification of individual agents as well as the general evaluative approach (Boffetta et al. 2009; Epidemiology Monitor 2012; Kabat 2012; McLaughlin et al. 2010, 2011). We discuss four of these criticisms below.

*Criticisms of epidemiology*. Some of the criticisms of the IARC process have occurred in the context of more general criticisms of epidemiology as a science (Kabat 2008); these were discussed in detail by Blair et al. (2009). Potential methodological weaknesses for observational epidemiologic studies are well recognized and can be found in any epidemiologic textbook (Checkoway et al. 2004; Rothman et al. 2008). Most studies are subject to one or more methodological limitations, but this does not necessarily invalidate their findings (Blair et al. 2009). In fact, the value of epidemiologic studies has been shown by the identification of a number of well-established human carcinogens, including tobacco, asbestos, benzene, hexavalent chromium, and some viruses, in multiple studies. Some critics also argue that small or nonexistent health risks are unjustifiably highlighted and hyped by researchers who have a vested interest in continued research funding and the need to publish to benefit their careers (Boffetta et al. 2008; Kabat 2008; McLaughlin et al. 2010, 2011; Taubes 1995). However, such overstated results are unlikely to exert much of an influence in a Monograph because IARC evaluations are based on the totality of the evidence. The problem would have to occur in multiple studies, and the Working Group would have to be unable to identify it or be unwilling to weigh such studies appropriately. Incorrect positive conclusions regarding carcinogenicity may also occur in reviews of multiple studies because of publication bias, which may selectively populate the literature only with “positive” findings. However, once a topic is recognized as scientifically important, reports on relevant studies will be published regardless of the findings, so publication bias is mainly a concern for newly arising issues. To evaluate the potential for publication bias, Working Groups consider whether stronger negative studies (both in terms of design and sample size) have emerged after publication of an initial cluster of smaller and/or weaker positive studies. Funnel plots help in the assessment of bias relating to sample size and publication bias (Borenstein et al. 2009). In contrast, there are no established statistical techniques to clearly characterize strength of design.

One of the distinctive features of epidemiology is that criticism and self-criticism are firmly embedded in the discipline. A great deal of work has been done on developing methods for critical appraisal (Elwood 2007) and for assessing the likely strength and direction of possible biases (Rothman et al. 2008). Epidemiologists and other members on Working Groups routinely use various approaches to assess possible bias in study design and analysis when weighing the strengths of different studies.

*The issue of false positives*. Epidemiology specifically has been criticized for a tendency to produce false-positive results (i.e., individual study associations not borne out by the weight of the evidence) or to preferentially report positive findings over negative or inconclusive findings (i.e., publication bias) (Boffetta et al. 2008, 2009; Ioannidis 2005; Kabat 2012; McLaughlin and Tarone 2013). This criticism has been most often applied to potential false positives from individual studies, but it has been inferred that this problem may also apply to overall hazard evaluations, which use findings from multiple studies. We will consider each of these issues in turn.

False-positive findings may occur by chance, particularly when many combinations of exposures and health outcomes have been examined in a single study without strong prior expectations of association; this happens often, for example, in genome-wide association studies where thousands of gene–disease associations are evaluated. Chance, of course, operates in all disciplines and in both observational and experimental studies. However, there are well-known statistical techniques to reduce the probability of declaring chance findings as “positive” (Rothman et al. 2008). Independent replication, however, is the most convincing way of checking for “chance” findings; hazard evaluations, such as those conducted by IARC Working Groups, rely heavily on reproducibility in independent studies and also interpret data following Bradford Hill principles (Hill 1965).

False negatives are more difficult to address, and perhaps they occur more frequently than false positives because of low statistical power, nondifferential misclassification of exposure and/or outcome, and incomplete follow-up, which tends to reduce the observed difference in risk between the exposed and nonexposed populations (Ahlbom et al. 1990; Blair et al. 2009; Grandjean 2005; Rothman et al. 2008). A new positive association stimulates research, whereas studies finding no associations tend to stifle further work.

There are difficulties in conducting epidemiologic studies of agents that are relatively “weak” carcinogens, or for stronger carcinogens where exposure is very low because bias and confounding can obscure weak positive associations (MacMahon et al. 1981). In general, weak carcinogens and low levels of exposure result in a smaller “signal-to-noise” ratio making the real signal more difficult to detect. Although the identification of small relative risks to humans poses special challenges to scientific research, the refinement of study designs, improvements in methods of exposure assessment, and the use of biomarkers have helped to address the problems (e.g., newer studies on the effects of air pollution, the growth in opportunities to examine gene–environment interactions) (Gallo et al. 2011). In some situations, there is less of a problem. For example, in occupational studies, exposures and relative risks may be higher while differences in lifestyle factors between different groups of workers are smaller (Checkoway et al. 2004); thus, any confounding by nonoccupational factors is likely to be weak, even from potent causes of cancer such as cigarette smoking (Siemiatycki et al. 1988). Of course, the interpretation of such studies is enhanced when there is supporting evidence from bioassays and/or mechanistic studies.

False-positive and false-negative findings in individual studies may arise by chance or bias, including bias due to confounding (Rothman et al. 2008). However, the evaluation of multiple independent epidemiologic studies from various geographic locations, involving a variety of study designs, as well as evidence from experimental studies, reduces the possibility that false-positive findings from any individual study influences the overall evaluation process. Some studies may have greater influence than others because of methodological strengths and/or large sample size. The use of information from a variety of study designs reduces the likelihood of false-positive evaluations because it is unlikely that the same biases will occur in multiple studies based on different populations under different study designs. Moreover, apparently conflicting results from epidemiologic studies do not necessarily indicate that some are false positive or false negative. This might, for example, reflect differences in levels of exposure or susceptibility to the effects of exposure (effect modification). Finally, judgment by the Working Group is not based exclusively on epidemiologic studies but usually also on results from laboratory and mechanistic studies that provide further evidence and biological coherence. For the Monographs that evaluate carcinogenic hazards associated with specific occupations or industries, the exposures of interest usually involve a complex mixture of chemicals. For these evaluations, most information comes from epidemiologic studies, although exposures to individual agents occurring at these workplaces may have been evaluated in experimental studies.

*Discontent with IARC Monograph processes*. The IARC Monograph evaluation process has been criticized and it has been alleged that “a number of scientists with direct experience of IARC have felt compelled to dissociate themselves from the agency’s approach to evaluating carcinogenic hazards” (Kabat 2012). This is a serious charge. However, the author of this claim provided no evidence to support the charge that a “number of scientists” have dissociated themselves from the process, nor has there been any indication of how many scientists have taken this step, or for what reason. In science, we expect sweeping statements such as this to be appropriately documented. We have not been able to identify any credible support for this contention.

There is an IARC Governing Council and a Scientific Council to provide oversight and guidance to the agency. The Governing Council represents the participating states and sets general IARC policy. It appoints the IARC Director and members of the Scientific Council. The latter are independent scientists who are selected to provide scientific expertise and not as representatives of the member states. They serve for 4 years and serve without pay. The voting members of Monograph Working Groups are not employed by IARC, and they perform this task without financial compensation. There have been 111 volumes, including six separate documents under Volume 100, and three Supplements. Over the years, as the number of publications for each agent to be evaluated increased, the size of Working Groups has increased. Early in the process they were sometimes as small as 10, but now they sometimes include as many as 30 scientists. We estimate that over the entire Monograph series, approximately 1,500 scientists have served as Working Group members, and of course many scientists have also served on the Advisory Groups, Scientific Council, and Governing Council. Thus, if even a small percentage of these scientists were disenchanted with the IARC process, it would result in a considerable number of such individuals and should be easy to document. To be taken seriously, the “dissociation” criticism needs to be supported by documented information describing the number of scientists who have taken this action.

*Criticisms of specific evaluations*. Some criticisms of the IARC process relate to specific agents, where it is asserted that the hazard evaluations of category 2B, 2A, or 1 are not supported by the scientific literature. In the 111 volumes of the Monographs produced over the four decades since 1971, 970 agents have been considered, 114 (12%) have been classified as carcinogenic to humans (Group 1), 69 (7%) as probably carcinogenic (Group 2A), 283 (29%) as possibly carcinogenic (Group 2B), 504 (52%) as not classifiable regarding their carcinogenicity (Group 3), and 1 (< 1%) as probably not carcinogenic to humans (Group 4). Thus, even for this highly select group of agents (i.e., those selected for evaluation because there was some concern that they might be carcinogenic), more than one-half were “not classifiable” or “probably not carcinogenic,” and a further 29% were placed into the category of possibly carcinogenic to humans. This distribution, based on nearly 1,000 evaluations in which fewer than one in five agents were classified as carcinogenic or probably carcinogenic to humans, does not support a conclusion that the process is heavily biased toward classifying agents as carcinogenic (Boffetta et al. 2009; Kabat 2012).

The monographs for formaldehyde, coffee, DDT, and radiofrequency electromagnetic radiation have been cited as examples of problematic evaluations by some (Kabat 2012) [among these, only formaldehyde was classified as known to be carcinogenic to humans (Group 1) by an IARC Working Group]. These are important agents. However, to accept the charge that IARC evaluations are fundamentally biased, one has to assume that the scientists who were members of the Working Groups were incapable of appropriately evaluating weaknesses in the data, or that they distorted the evaluative process because of personal biases. In our experience, neither of these assertions is correct. Dissent among scientists is not unusual in any area of science. It is a strength of the scientific process. The IARC process capitalizes on this by bringing scientists from different disciplines together in one room to evaluate the literature and to reach a reasoned conclusion. Differences of opinion occur among Working Group members. These differences, however, typically involve disputes related to assignment to adjacent classification categories. It is instructive that there are no instances in which a carcinogen classified at the Group 1 level by one Working Group has been reversed by another. The recent review of all Group 1 agents for Volume 100 provided ample opportunity to reverse such previous classifications, but none occurred. Every scientist could probably name a substance that has been reviewed by IARC that they might personally place in a different category from that assigned by the Working Group, but this is one opinion against the collective wisdom and process of the Working Group.

*Criticisms of the composition of the working groups*. The composition of the Working Groups has also been criticized (Erren 2011; McLaughlin et al. 2010, 2011); it has been argued that members of the Working Groups who have conducted research on the agents under evaluation have a vested interest in advancing their own research results in the deliberations. This criticism has been addressed directly by Wild and colleagues (Wild and Cogliano 2011; Wild and Straif 2011) from IARC, and we know of no evidence to support this contention. Even if some scientists on the Working Group have performed research on some of the agents being considered, they make up a minority of the Working Group because several agents are usually evaluated in a single meeting, so the number of Working Group members who have conducted research on any one agent is typically small. Our experience has been that having some scientists who are knowledgeable about the studies of the agent under evaluation (and can therefore answer technical queries) and others from different, but related, fields provides a knowledgeable and balanced mix of scientific backgrounds for a thoughtful evaluation of the literature.

Working Group members do not receive any fee for their work, but they are paid travel expenses, and there is some prestige associated with service on an IARC Monograph. However, most scientists asked to serve on IARC Working Groups have already achieved some measure of scientific stature, and there is no reason why this should bias their evaluation in one direction or the other. In addition, IARC strictly requires that any conflict of interests be divulged, and does not allow those with conflicts of interest to serve on Working Groups, although nonvoting observers who may have conflicts of interest are able to attend the Working Group meetings.

## Conclusions

For more than four decades the IARC Monograph Programme has provided evaluations of cancer hazards to humans from many different exposures and agents. These are often the first evaluations of new and emerging threats to public health and, consequently, are subject to intense scrutiny. Although these evaluations are widely respected and used by many organizations, institutions, companies, and government agencies to improve the public’s health, IARC has recently been subject to criticism over conclusions on specific agents, the process that leads to such conclusions, and membership of the Working Groups. Debate and criticism facilitate self-correction and a check on the validity in science. We are concerned, however, that the criticisms expressed by a vocal minority regarding the evaluations of a few agents may promote the denigration of a process that has served the public and public health well for many decades for reasons that are not supported by data.

There has been very broad involvement of the scientific community in the IARC Monograph Programme through participation in the Working Groups and service on the IARC Governing and Scientific Councils and ad hoc Advisory Board for the Monograph Programme. The long list of scientists who are coauthors of this paper attests to the strong support that IARC has in the scientific community. Many exposures that IARC has evaluated have also been independently evaluated by other institutions, such as the U.S. National Toxicology Program (https://ntp.niehs.nih.gov/); U.S. Environmental Protection Agency (http://www.epa.gov/); National Academy of Sciences (http://www.nasonline.org/); the American Conference of Governmental Industrial Hygienists (ACGIH) Threshold Limit Values and Biological Exposure Indices (http://www.acgih.org/); the Nordic Expert Group for Criteria Documentation of Health Risks from Chemicals (http://www.av.se/arkiv/neg/); Institute of Occupational Medicine (http://www.iom-world.org/); World Cancer Research Fund/American Institute for Cancer Research (WCRF/AICR) Expert Reports; European Chemicals Agency (https://echa.europa.eu); Swedish Criteria Group for Occupational Standards (2013); California Office of Environmental Hazard Assessment (Proposition 65; http://oehha.ca.gov/prop65/background/p65plain.html); Health Canada Bureau of Chemical Safety (http://www.hc-sc.gc.ca/ahc-asc/branch-dirgen/hpfb-dgpsa/fd-da/bcs-bsc/index-eng.php); Scientific Committee on Occupational Exposure Limits (SCOEL), European Commission, Employment, Social Affairs and Inclusion (http://ec.europa.eu/social/main.jsp?catId=148&langId=en&intPageId=684); European Food Safety Authority (EFSA 2013); and European Chemicals Agency (ECHA; http://echa.europa.eu/). Assessments from these groups typically come to conclusions similar to those from IARC. This further indicates broad agreement within the scientific community regarding evidence on carcinogenicity in the scientific literature and expands the number of scientists who do not have a “vested interest” but who have generally agreed with those conclusions.

Disagreement with the conclusions in an IARC Monograph for an individual agent is not evidence for a failed or biased approach. Some disagreement about the carcinogenic hazard of important agents seems inherent to the scientific enterprise and is unavoidable at early stages of the hazard evaluation, where IARC usually operates. Because the evaluations are not—and should not be—static, it is difficult to see how such assessments could be addressed any differently. Substances now universally recognized as human carcinogens (e.g., tobacco, asbestos) at one time went through a quite lengthy period of contentious debate (Michaels 2006, 2008). Any process can in theory be improved with fair and constructive criticism; appropriate reviews may take place from time to time, and we would support continued review and improvement of the IARC processes. However, as a group of international scientists, we have looked carefully at the recent charges of flaws and bias in the hazard evaluations by IARC Working Groups, and we have concluded that the recent criticisms are unfair and unconstructive.

**Appendix 1: Classification Categories for the Overall Evaluation for the IARC Monographs (IARC 2006)**

**Group 1: The agent is *carcinogenic to human*s.**

This category is used when there is sufficient evidence of carcinogenicity in humans. Exceptionally, an agent may be placed in this category when evidence of carcinogenicity in humans is less than sufficient but there is sufficient evidence of carcinogenicity in experimental animals and strong evidence in exposed humans that the agent acts through a relevant mechanism of carcinogenicity.

**Group 2.**

This category includes agents for which, at one extreme, the degree of evidence of carcinogenicity in humans is almost *sufficient*, as well as those for which, at the other extreme, there are no human data but for which there is evidence of carcinogenicity in experimental animals. Agents are assigned to either Group 2A (*probably carcinogenic to humans*) or Group 2B (*possibly carcinogenic to humans*) on the basis of epidemiological and experimental evidence of carcinogenicity and mechanistic and other relevant data. The terms *probably carcinogenic* and *possibly carcinogenic* have no quantitative significance and are used simply as descriptors of different levels of evidence of human carcinogenicity, with *probably carcinogenic* signifying a higher level of evidence than *possibly carcinogenic*.

**Group 2A: The agent is *probably carcinogenic to humans.***

This category is used when there is *limited evidence of carcinogenicity* in humans and *sufficient evidence of carcinogenicity* in experimental animals. In some cases, an agent may be classified in this category when there is *inadequate evidence of carcinogenicity* in humans and *sufficient evidence of carcinogenicity* in experimental animals and strong evidence that the carcinogenesis is mediated by a mechanism that also operates in humans. Exceptionally, an agent may be classified in this category solely on the basis of *limited evidence of carcinogenicity* in humans. An agent may be assigned to this category if it clearly belongs, based on mechanistic considerations, to a class of agents for which one or more members have been classified in Group 1 or Group 2A.

**Group 2B: The agent is *possibly carcinogenic to humans*.**

This category is used for agents for which there is *limited evidence of carcinogenicity* in humans and less than *sufficient evidence of carcinogenicity* in experimental animals. It may also be used when there is *inadequate evidence of carcinogenicity* in humans but there is *sufficient evidence of carcinogenicity* in experimental animals. In some instances, an agent for which there is *inadequate evidence of carcinogenicity* in humans and less than *sufficient evidence of carcinogenicity* in experimental animals together with supporting evidence from mechanistic and other relevant data may be placed in this group. An agent may be classified in this category solely on the basis of strong evidence from mechanistic and other relevant data.

**Group 3: The agent is *not classifiable as to its carcinogenicity to humans*.**

This category is used most commonly for agents for which the evidence of carcinogenicity is *inadequate* in humans and *inadequate* or *limited* in experimental animals.

Exceptionally, agents for which the evidence of carcinogenicity is *inadequate* in humans but *sufficient* in experimental animals may be placed in this category when there is strong evidence that the mechanism of carcinogenicity in experimental animals does not operate in humans.

Agents that do not fall into any other group are also placed in this category.

An evaluation in Group 3 is not a determination of noncarcinogenicity or overall safety. It often means that further research is needed, especially when exposures are widespread or the cancer data are consistent with differing interpretations.

**Group 4: The agent is *probably not carcinogenic to humans*.**

This category is used for agents for which there is *evidence suggesting lack of carcinogenicity* in humans and in experimental animals. In some instances, agents for which there is *inadequate evidence of carcinogenicity* in humans but *evidence suggesting lack of carcinogenicity* in experimental animals, consistently and strongly supported by a broad range of mechanistic and other relevant data, may be classified in this group.
